# Identifying target populations to align with decision-makers’ needs

**DOI:** 10.1093/aje/kwae129

**Published:** 2024-06-19

**Authors:** Jennifer L Lund, Anthony A Matthews

**Affiliations:** Department of Epidemiology, Gillings School of Global Public Health, University of North Carolina at Chapel Hill, Chapel Hill, NC 27599, United States; Unit of Epidemiology, Institute of Environmental Medicine, Karolinska Institutet, SE-171 77 Stockholm, Sweden

**Keywords:** target populations, decision-makers, causal inference

## Abstract

Randomized trials estimate the average treatment effect within individuals who are eligible, invited, and agree to enroll. However, decision-makers often require evidence that extends beyond the trial's enrolled population to inform policy or actions for their specific target population. Each decision-maker has distinct target populations, the composition of which may not often align with that of the trial population. As researchers, we should identify a decision-maker for whom we aim to generate evidence early in the research process. We can then specify a target population of their interest and determine if a policy or action can be informed using results from a trial alone, or if additional complementary real-world data and analysis are required. In this commentary, we outline 5 key groupings of decision-makers: policymakers, payers, purchasers, providers, and patients. We then specify relevant target populations for decision-makers interested in the effectiveness of beta-blockers after a myocardial infarction with preserved ejection fraction. Finally, we summarize the scenarios in which results from a randomized trial may or may not apply to these target populations and suggest relevant analytic approaches that can generate evidence to better align with a decision-maker’s needs.

**This article is part of a Special Collection on Pharmacoepidemiology**.

***Editor’s note:** The opinions expressed in this article are those of the authors and do not necessarily reflect the views of the *American Journal of Epidemiology.

## Introduction

For any question on the effectiveness or safety of a medical treatment, a randomized trial estimates the average treatment effect in a population who are eligible, invited, and agree to enroll in the trial. However, when a decision-maker needs to use results reported in a trial to inform a policy or action, the population who enrolled in the trial often does not align with the target population relevant to their needs. The target population is defined by the overall group of individuals who would be affected by the policy or action put in place by the decision-maker.

As researchers interested in generating actionable evidence, identification of a decision-maker we aim to inform means we can clearly specify their target population. We are then better positioned to decide whether decisions or policies for this target population can be made using trial results alone, or if additional complementary data and analysis are required produce valid and impactful evidence.

In this commentary, we outline 5 key decision-makers who use results of randomized trials, describe the general makeup of their target populations, then use a motivating example of the effectiveness of beta-blockers after a myocardial infarction with preserved ejection fraction to specify the target populations for several relevant decision-makers. Finally, we briefly highlight methods that leverage real-world data when the estimate reported in a trial may not directly apply to a target population of interest.

## Target populations for decision-makers using randomized trial evidence

There are several distinct groups involved in medical decision-making: policymakers, payers, purchasers, providers, and patients.^1^^,^^2^ Policymakers, including regulators (eg, the US Food and Drug Administration) and clinical guideline–making entities (eg, the American College of Cardiology), make decisions about marketing approvals for a treatment or clinical recommendations about a treatment to health care providers. They often are interested in target populations that include individuals likely eligible for treatment based on existing trial evidence. Payers include both public (eg, Medicare) and private (eg, United Healthcare) entities that make coverage decisions about the types of treatment they will make available to individuals covered by their health plan (eg, beneficiaries). Their target population of interest includes individuals within their health plan who may receive treatment. Purchasers provide or deliver health care, such as health care systems (eg, University of North Carolina [UNC] Health) and focus on the delivery of health care to patients. These entities are interested in the specific patient population who are cared for at their facilities as their target population of interest. Providers include an array of health care professionals (eg, primary care, specialists, pharmacists, nurses) who decide whether to recommend and administer a treatment to a patient. These providers are often interested in the patient at hand, but also potentially are interested in a broader population of patients who they typically see in their practice as the target population of interest. Patients and their caregivers are the final decision-makers who must ultimately agree to a plan of treatment with their provider. Patients are primarily concerned with themselves as the target population of interest; however, patients may also be concerned about how the effectiveness and safety of a treatment they receive affects their families. For example, they may not be willing to accept a greater risk of side effects for a small gain in risk of survival, if having such side effects would require additional familial support.

## Motivating example: effectiveness of beta-blockers after a myocardial infarction with preserved ejection fraction

The absence of clear randomized trial evidence on the effectiveness of beta-blockers in patients with acute myocardial infarction with preserved systolic ejection fraction has resulted in general uncertainty of how to treat patients. The ongoing Randomized Evaluation of Decreased Usage of Beta-Blockers After Acute Myocardial Infarction (REDUCE-AMI) trial aims to address this gap and determine whether treatment with long-term oral beta-blockers in patients with an acute myocardial infarction and preserved left ventricular ejection fraction reduces the composite endpoint of death of any cause or recurrent myocardial infarction.^3^ The REDUCE-AMI trial is a registry-based, prospective, open-labeled, parallel randomized trial conducted in Sweden (*n* = 36 centers), Estonia (*n* = 1 center), and New Zealand (*n* = 6 centers). Individuals are eligible if they are older than 18 years; within 1 to 7 days after a type 1 myocardial infarction; have undergone coronary angiography that documented obstructive coronary artery disease; have echocardiography results showing a normal ejection fraction, defined as ejection fraction ≥50%; and consent to enroll. Exclusion criteria are any condition that may influence an individual’s ability to comply with study protocols (eg, a diagnosis of dementia or psychological disorders), contraindications for beta-blockers, or indication for beta-blockers other than secondary prevention.

The REDUCE-AMI trial is a large, pragmatic, randomized trial. But the population who enroll in the trial may not represent characteristic distribution of other target populations. First, like all clinical trials, individuals must consent to enroll and participate, and it is well known that those who enroll in trials are generally younger and healthier than those who do not enroll. Second, the geographical regions in which the REDUCE-AMI trial is recruiting are not necessarily clinically, demographically, or socially representative of other geographic locations. Third, the REDUCE-AMI trial excludes individuals who could receive beta-blockers in routine care (ie, those with conditions that may influence their ability to comply with the study protocol). Finally, engagement effects (ie, Hawthorne effects) may alter the effects observed in trial settings.

In Table 1, we identify 5 decision-makers that could be interested in the results of the REDUCE-AMI trial and specify 1 potential target population for each decision-maker. The population that will enroll in the REDUCE-AMI trial does not align with the composition of any of our 5 potential target populations.

**Table 1:** Decision-makers interested in the results of the Randomized Evaluation of Decreased Usage of Beta-Blockers After Acute Myocardial Infarction trial and their target populations.

**Decision-maker**	**Example**	**Target population**
Policymakers	American College of Cardiology/American Heart Association	Everyone in the United States with a myocardial infarction and preserved ejection fraction
Payers	Medicare	Medicare beneficiaries in the United States (those who are either older than 65 years, have end-stage renal disease, or other qualifying disabilities) with a myocardial infarction and preserved ejection fraction
Purchasers	UNC Health	Everyone admitted to 1 of the hospitals within the UNC health care system with a myocardial infarction and preserved ejection fraction
Providers	Cardiologist working within a UNC outpatient practice	Everyone seen by the cardiologist within the outpatient practice with a myocardial infarction and preserved ejection fraction OR an individual patient admitted under the care of the cardiologist for the same reason
Patients	Patient admitted to a UNC medical center	An individual patient admitted to a UNC medical center with a myocardial infarction and preserved ejection fraction

Abbreviation: UNC, University of North Carolina.

## When do results reported in trials apply to other target populations?

A treatment effect estimated in a randomized trial will not be the same as the treatment effect in another target population if there is any treatment-effect measure modification, known or unknown; and the distribution of the modifier(s) differs between the trial and target populations. Conversely, if there is no treatment-effect measure modification or effect modifiers are equally distributed between the trial and target populations, then the effect estimated in the randomized trial will be the same as the treatment effect in the target population, within sampling variability. The assumption of no treatment-effect measure modification is, however, often implausible and hard to verify, given trials are only powered to estimate an average effect in the enrolled population. The likelihood of equally distributed effect modifiers is also a strong assumption, given the general lack of representativeness of those who enroll in trials.

The results of the REDUCE-AMI trial, therefore, will not directly extrapolate to the aforementioned target populations if any covariate that is unbalanced between the trial and target population also modifies the effect of beta-blockers. If true, the question then becomes, if the trial had recruited a population that was representative of a target population of interest, would the result be materially different from the effect estimated in the trial? And would the new result convince the decision-maker to implement a different policy or action? This dilemma can only be speculated on, without additional analysis and real-world data from the target population.

## Methods to generate evidence to complement results from randomized trials

There are several methodological approaches that can be used to estimate treatment effects in alternative target populations. Increasingly, researchers are interested in extending inferences (eg, generalizing or transporting) from randomized trials to a target population, which requires combining treatment, outcome, and baseline covariate data from a trial with baseline covariate data from an observational data set.^4–8^ Of note, implementation of these methods requires baseline covariate data from individuals in both the trial and target population, which motivates the need for trial investigators to collect data on potential treatment-effect measure modifiers. These methods may be of particular interest to policymakers, payers, and purchasers, because they generate average treatment-effect estimates that could be more relevant to the target populations they are interested in.

Methods to extend inferences require 2 strong assumptions, in addition to those needed to make inference in a trial: namely, conditional exchangeability between the trial and target population and positivity of trial participation.^6^ Conditional exchangeability requires that participants in the trial are exchangeable with (ie, similar to) individuals in the target population, conditional on baseline effect measure modifiers. For example, if we believe age is the only important effect measure modifier in the REDUCE-AMI trial, and the distribution of age differs between the trial and target population, we can achieve conditional exchangeability when using these methods if we have collected data on age in both trial and target populations. Positivity of trial participation requires that for each individual in the target population, there is a non-zero probability of being represented in the trial (with respect to the effect measure modifiers on the scale of interest). As such, if people with dementia were entirely excluded from the REDUCE-AMI trial but are included in your target population, the methods to extend trial inferences to target populations may not be applied without further conditional exchangeability assumptions. The same conditional exchangeability assumptions would apply to differences in geography, for example, if the trial were conducted in Sweden and the target population were in the United States. In this setting, we would have to assume that, conditional on other effect measure modifiers, that geography (or its proxies, like health care system generosity) do not independently modify the effects of treatment.

If these assumptions are not met, a potential alternative is to conduct a traditional observational study that directly estimates the treatment effect within the target population of interest (eg, including those systematically excluded from the trial). As with all observational studies seeking to make causal inferences, valid treatment-effect estimation requires assumptions including internal exchangeability (ie, no unmeasured confounders and no informative censoring), positivity (ie, nonzero probability of exposure for every combination of values of exposure and confounders in the population), and consistency (ie, an individual’s potential outcome under a well-defined exposure is precisely the observed outcome).^9^ A more practical issue in conducting an observational analysis is that of data availability. For example, a researcher cannot study the effect of a newly marketed treatment until a sufficient number of individuals in the target population have used the treatment, which may require some time after the approval and launch of the treatment.

Finally, providers and patients are often most interested in estimating individualized treatment effects. The methods we have described estimate population average treatment effects and thus cannot estimate the anticipated treatment effect for a given individual. New and emerging methods that use counterfactual clinical prediction models may provide a pathway to infer individualized treatment effects to target patients who may benefit most.^10^^,^^11^

## Conclusion

As researchers, we traditionally identify an interesting clinical question; undertake a study to answer that question in a convenient population, the makeup of whom is usually driven by data availability, then identify a decision-maker who is most likely to use the results. A more efficient approach is to identify a decision-maker for whom we want to generate evidence at the same time as choosing the clinical question; we can then clearly specify a target population of interest for this decision-maker and identify appropriate data and methods. In this commentary, we have identified 5 groups of decision-makers to consider when designing studies on treatment effectiveness and safety. Going forward, researchers can use this structure to generate evidence aligned with decision-makers’ needs.
